# Supplementary material for: Time-Course Changes of Extracellular Matrix Encoding Genes Expression Level in the Spinal Cord Following Contusion Injury—A Data-Driven Approach
Source: Int J Mol Sci. 2021 Feb 9;22(4):1744. doi: 10.3390/ijms22041744 (PMC7916102; doi:10.3390/ijms22041744)
Supplement: Supplementary file 1 [file ijms-22-01744-s001.pdf]

International Journal of
*Molecular Sciences*

MDPI

**Supplementary Figure S1**

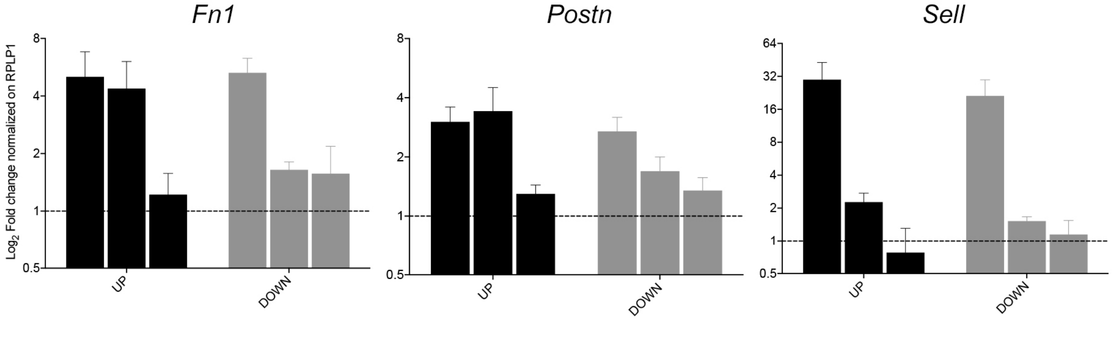


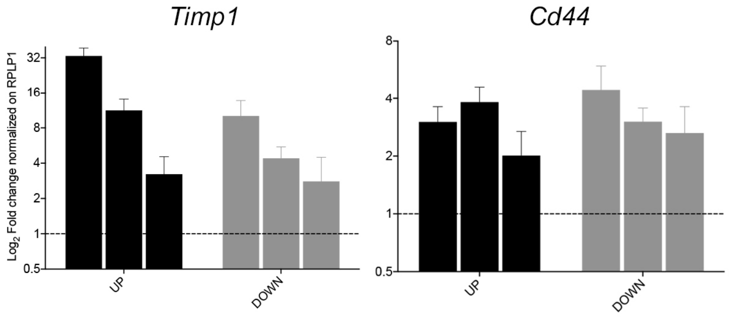


| | PCR array | qPCR (AVG) |
|---|---|---|
| *Fn1* - UP - 1 DPL | +3.46 | 5.04 |
| *Fn1* - UP - 8 DPL | +3.19 | 4.37 |
| *Postn* - UP - 1 DPL | +3.06 | 3.01 |
| *Postn* - UP - 8 DPL | +5.06 | 3.41 |
| *Sell* - UP - 1 DPL | +22.03 | 29.88 |
| *Sell* - UP - 8 DPL | +2.14 | 2.28 |
| *Sell* - DOWN - 1 DPL | +7.99 | 21.20 |
| *Cd44* - UP - 1 DPL | +4.96 | 3.01 |
| *Cd44* - UP - 8 DPL | +4.27 | 3.82 |
| *Cd44* - DOWN - 1 DPL | +2.31 | 4.44 |
| *Timp1* - UP - 1 DPL | +28.57 | 33.21 |
| *Timp1* - UP - 8 DPL | +12.40 | 11.28 |
| *Timp1* - UP - 45 DPL | +2.32 | 3.22 |
| *Timp1* - DOWN - 1 DPL | +13.95 | 10.10 |
| *Timp1* - DOWN - 8 DPL | +5.22 | 4.40 |
| *Timp1* - DOWN - 45 DPL | +2.29 | 2.80 |

**Supplementary Figure S2**

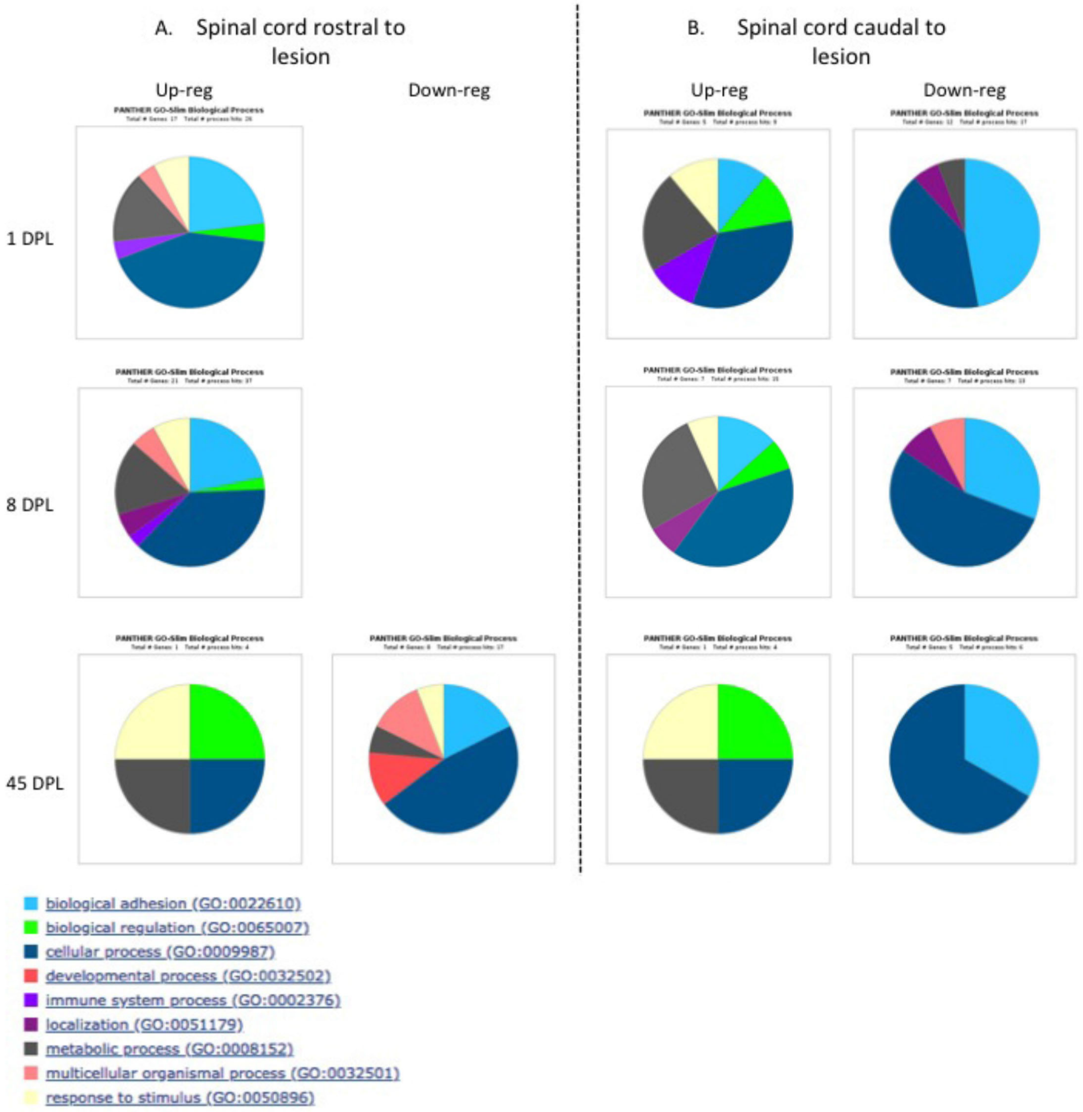

**Supplementary Figure S3**

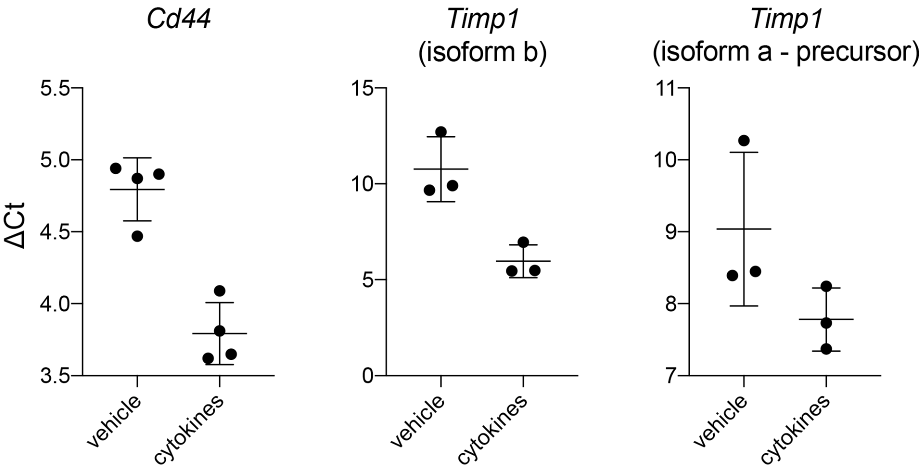

**Supplementary Table S1**

| Gene | Correlation *p* value (Pearson r) |
|---|---|
| *Cd44* (caudal) | 0.7085 |
| *Cd44* (rostral) | 0.2032 |
| *Fn1* (caudal) | 0.8380 |
| *Fn1* (rostral) | 0.5819 |
| *Postn* (caudal) | 0.8852 |
| *Postn* (rostral) | 0.8590 |
| *Sell* (caudal) | 0.7691 |
| *Sell* (rostral) | 0.5046 |
| *Tnc* (caudal) | 0.2912 |
| *Tnc* (rostral) | 0.5474 |
| *Acan* (caudal) | 0.5013 |
| *Acan* (rostral) | 0.9927 |
| *Tnr* (caudal) | 0.4781 |
| *Tnr* (rostral) | 0.8055 |
| *Cspg4* (caudal) | 0.7554 |
| *Cspg4* (rostral) | 0.6695 |
| *Slit1* (caudal) | 0.6475 |
| *Slit1* (rostral) | 0.6917 |
| *Slit2* (caudal) | 0.6471 |
| *Slit2* (rostral) | 0.8710 |
